# Impact of moral education on anomie behaviors in university students’ physical education classes: serial mediation of attitudes toward sports norms and self-efficacy

**DOI:** 10.1186/s40359-025-03867-7

**Published:** 2025-12-22

**Authors:** Liping Liu, Yifei Song, Han Liu, Shanping Chen, Yao Shang, Zhongjiang Zhang

**Affiliations:** School of Physical Education, Xi’an Jiaotong University, Xi’an, 710049 China

**Keywords:** Moral education, Anomie behavior, Attitudes toward normative behaviors, Self-efficacy, University students

## Abstract

**Background:**

Against the backdrop of building an education powerhouse, this study aims to explore the relationship between moral education and anomie behaviors in university physical education classes, and to examine the serial mediating role of attitudes toward sports norms and self-efficacy in this relationship.

**Methods:**

A questionnaire survey was conducted among 2,340 undergraduate students from 20 universities across the country. The collected data were organized and analyzed using SPSS Statistics 27.0.

**Results:**

Moral education exerted a significant negative predictive effect on anomie behaviors in physical education classes. This influence was primarily mediated through the serial mediation pathway of attitudes toward sports norms and self-efficacy. The effect sizes for these three pathways were -0.051, -0.018, and -0.013, accounting for 45.54%, 16.07%, and 11.61% of the total effect, respectively.

**Conclusion:**

Moral education directly influences anomie behaviors in university physical education classes. Attitudes toward sports norms and self-efficacy for sports-related norms play a serial mediating role in the relationship between moral education and anomie behaviors in physical education classes.

**Supplementary Information:**

The online version contains supplementary material available at 10.1186/s40359-025-03867-7.

## Introduction

The Outline of the Plan for Building an Education Powerhouse (2024–2035) explicitly states the need to “strengthen and improve ideological and political education in schools in the new era,” emphasizing the importance of “promoting students” healthy growth and all-around development.’ Against this backdrop, the educational role of physical education courses in higher education has been elevated to an unprecedented level. However, during the process of physical education instruction, students frequently exhibit anomie behaviors such as tardiness, leaving early, failing to observe classroom discipline, and lacking active participation in physical activities. These behaviors not only undermine the quality and effectiveness of physical education teaching but also severely constrain the fulfillment of the course’s educational mission. Therefore, exploring effective strategies to prevent such anomie behaviors in university physical education has become an urgent and vital issue in sports teaching and management.

In recent years, research on anomie behaviors in university sports has gradually gained momentum. Existing studies have created psychological models to explain the mechanisms behind such anomie behaviors among university students, exploring the roles of motivation, attitudes, and self-efficacy in shaping these patterns [1]. However, empirical research remains limited, especially lacking in a detailed analysis of the full underlying mechanisms. Although existing studies have examined the impact of external social support factors on anomie behaviors in university sports through psychological motivation [2, 3], these investigations have limitations, particularly in the depth and scope of their explanations. Conversely, research on anomie behaviors among ordinary university students in physical education classrooms remains relatively limited. Chen Shanping laid important groundwork for quantitative studies by establishing a scientific classification and theoretical framework for anomie behaviors in university sports [4]. However, studies exploring the dimensions of such anomie behaviors in university physical education classes are still few, and comprehensive analyses of the multiple factors influencing this behavior are even rarer. Additionally, in research on moral education, Zhang Zhongjiang emphasized the macro-level importance of physical education in moral development, yet did not clarify the specific pathways and mediating processes through which it affects students’ behavioral choices via intrinsic psychological mechanisms [5]. Wei Xin and Xu Fang [6], Yang Xiangquan [7], and other scholars confirmed the positive influence of physical education courses in fostering students’ sense of norms and ethical behavior. Notably, particularly within the international academic community, no direct empirical tests have yet been conducted on the potential chain-mediated effects of attitudes toward sports norms and self-efficacy in the relationship between moral education and anomie behaviors in university students’ physical education. Therefore, exploring the intrinsic pathways and enabling conditions through which moral education influences anomie behaviors in university students’ physical education by leveraging psychological mechanisms remains an important area for further research.

Based on this, This study creatively develops the “attitudes toward sports norms and self-efficacy—anomie behaviors in university students’ physical education”. This model not only the theoretical framework and practical effectiveness of physical education’s educational function but also provides empirical evidence and operational guidance for universities to optimize physical education strategies and enhance educational quality.

### Literature review and research hypotheses

#### The direct impact of moral education on anomie behaviors in physical education classes

Kohlberg’s theory of moral cognitive development posits that moral judgment is the most fundamental factor determining students’ ethical behavior, and the formation of moral judgment is closely intertwined with social practice [8]. Durkheim’s theory of social disorganization argues that the breakdown of social norms’ constraints on individual behavior leads to social disorder and deviant conduct [9]. Behaviors such as violating classroom discipline and cheating on exams in physical education classes are precisely the micro-level manifestations of Durkheim’s theory of social disorganization. These actions are directly driven by the individual’s “moral judgment,” as emphasized by Kohlberg. Traditional views on sports ethics education claim that sports ethics include virtuous qualities students should embody during sports activities, such as fair competition, honesty, integrity, courageous effort, and harmonious camaraderie [10]. Anomie behaviors in sports refers to actions that violate social norms, including sports ethics, competition rules, regulations, and sports laws during participation. Anomie behaviors in university students’ physical education classes encompasses four key areas: violations of classroom discipline, breaches of examination protocols, infractions of sports ethics, and violations of venue regulations [4]. Research shows that moral education can turn ethical principles and norms into participants’ internal convictions, helping them develop strong moral concepts [11]. By consciously cultivating a moral environment in the classroom, teachers can exert direct influence to restrain and guide students’ anomie behaviors. International research similarly indicates that fostering a positive moral environment can effectively curb students’ anomie behaviors [12, 13].

Conversely, physical education classes lacking moral education may encourage anomie behaviors. The absence of moral education during athlete development significantly contributes to the rise of moral anomie behaviors [36]. Without moral education, some stakeholders may ignore breaches of sporting ethics, even taking pride in profiting through improper means. Additionally, students might prioritize winning at all costs due to a lack of moral guidance. This can result in the use of unscrupulous tactics to win or escalating competitive conflicts due to a misunderstanding of the value of “friendly competition” [14, 15, 16]. Based on the analysis above, this study proposes the following research hypothesis.

H1: Moral education significantly reduces anomie behaviors in university physical education classes.

#### The mediating role of attitudes toward sports norms

According to the Theory of Planned Behavior [17], the more positive an individual’s attitude toward a certain behavior, the stronger their behavioral intention becomes, and the more likely that behavior is to occur. In physical education settings, moral education helps students develop positive attitudes toward sports norms by systematically explaining their value and analyzing typical cases. When students genuinely recognize the significance of adhering to sports norms, their willingness to comply significantly increases, thereby reducing the occurrence of anomie behaviors in university students’ physical education.Attitudes toward sports norms refer to university students’ value judgments regarding the consequences of adhering to or violating sports behavioral norms. These include assessments of outcomes related to both anomie behaviors and normative behaviors., with the value system covering both beneficial and harmful aspects [4]. Effective moral education can promote positive attitudes toward sports norms. When university students fully understand the importance and value of these norms through such education—recognizing their role in maintaining orderly sports activities and supporting personal growth—they are more likely to voluntarily follow and uphold these standards, willingly accepting their evaluation [18]. This positive attitude encourages them to internalize self-control over anomie behaviors in practice [19], thereby strengthening their attitude toward sports norms.

Regarding the psychological mechanisms behind anomie behaviors in sports, attitudes toward sports norms conduct determine individuals’ intentions to engage in anomie behaviors in sports [1, 20]. International research similarly indicates that normative behavioral attitudes can directly predict cheating behavior [21], while mediating between cognition and behavioral intent [22]. Based on this analysis, this study proposes the following research hypothesis.

H2: Attitudes toward sports norms mediate the link between moral education and anomie behaviors in university physical education classes.

#### Hypothesis on the mediating role of self-efficacy in sports norms

Bandura’s self-efficacy theory posits that an individual’s subjective judgment regarding their ability to successfully perform a behavior influences their motivation and effort level [23]. In physical education classroom settings, moral education can significantly enhance students’ belief in adhering to sports norms by explaining the value of these norms, providing exemplary models, and creating successful experiences of compliance. Self-efficacy in sports norms represents students’ belief in their ability to successfully adhere to various sports norms. The strength of this belief directly determines their behavioral performance during physical activities. Self-efficacy in sports norms refers to the degree of confidence university students have in complying with various sporting regulations during physical activities [4]. Effective moral education may foster higher levels of self-efficacy in sports norms [24, 25]. Ethical education emphasises cultivating awareness of rules [26]. When students perceive the value and significance of rules through such education, they become more inclined to believe they can uphold normative requirements during physical education classes. This confidence reinforces their conviction to adhere to sports norms, thereby elevating their self-efficacy in sports norms. An individual’s psychological disposition regarding their capacity to adhere to physical education norms (confidence or lack there of) influences behaviors.

Regarding the psychological mechanisms behind anomie behaviors in university students’ physical education classes, higher self-efficacy related to these norms correlates with a greater willingness to comply voluntarily, thus decreasing anomie behaviors in sports [27]. International research similarly indicates that high self-efficacy significantly enhances the conversion from cognition to behavior [28].Conversely, individuals with low self-efficacy tend to engage in more non-compliant behaviors, such as superficial efforts or even rule evasion through disobedience [29]. Therefore, self-efficacy plays a role in transforming moral cognition into moral action, with morality shaping conduct through self-efficacy [27]. Based on this analysis, this study proposes the following research hypotheses.

H3: Self-Efficacy in Sports Norms mediates the relationship between moral education and anomie behaviors in university physical education classes.

#### Hypothesis of a serial mediation through attitudes toward sports norms and self-efficacy

According to Bandura’s social cognitive theory [30], the environment, the individual, and behavior engage in dynamic interactions within a triadic interaction model. In the field of physical education, moral education, as a key environmental factor, may shape students’ attitudes toward sports norms, thereby enhancing their self-efficacy in sports norms. This ultimately establishes a complete psychological pathway leading to a reduction in anomie behaviors in sports. Moral education can effectively shape university students’ positive attitudes toward sports norms. By constructing a multidimensional repository of academic anomie cases [31], moral education provides students with behavioral norms and value standards in a vivid and accessible manner [32]. This enables them to cognitively grasp the significance and value of adhering to sports norms, fostering emotional identification and attitudinal inclination towards practising normative behaviors. Such positive attitudes toward sports norms, in turn, significantly enhance self-efficacy in adhering to them. Existing research indicates that a positive attitude towards seeking medical care enhances patients’ self-efficacy in healthcare utilization and reduces the occurrence of delayed medical seeking [33].

**Fig. 1:**
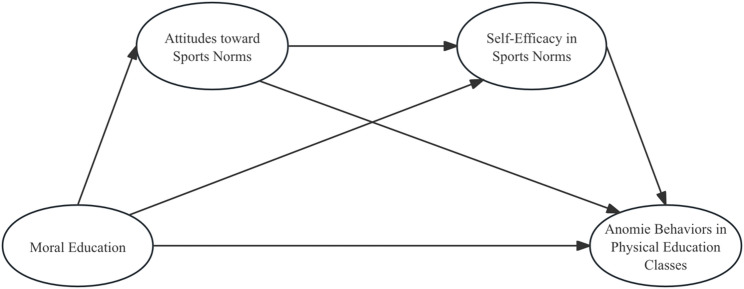
Analytical framework

When students hold an accepting and supportive behavioral attitude towards adhering to sports norms, they become more confident in engaging in normative practices. Self-efficacy in sports norms further influences anomie behaviors in university physical education classes. Bandura notes that individuals with high self-efficacy are more likely to transition from ‘knowledge acquisition’ to ‘behavioral implementation,’ tending to adopt and persist in healthy behaviors [34]. Their confidence in their own capabilities enables them to overcome obstacles in behavioral execution, thereby reducing the occurrence of anomie behaviors. Based on the above analysis, this study proposes the following hypothesis:

H4: Attitudes toward sports norms and self-efficacy exert a serial mediation effect on the influence of moral education on anomie behaviors in university physical education classes. The hypothetical framework for each variable is illustrated in Fig. 1.

## Research subjects and methods

### Research subjects

The data utilized in this study originate from Professor Chen Shanping’s National Social Science Fund project approved in 2017. Conducted between January and November 2018, the project implemented three distinct data collection phases: an open-ended questionnaire survey, a closed-ended pre-survey, and a nationwide closed-ended formal survey, tailored to the requirements of each research stage. The analysis employs data from the nationwide closed formal survey. Sample selection was designed based on two dimensions: representativeness and survey feasibility. To ensure representativeness, 20 higher education institutions were randomly selected across six regions: four in North China, two in Northeast China, five in East China, four in Central and South China, three in Southwest China, and two in Northwest China. The survey targeted undergraduate students enrolled at these institutions. Considering survey feasibility, stratified random sampling was employed to select respondents. From each of the 20 institutions, 120 students were recruited. To ensure balanced distribution across year groups and genders, collaborating staff at each university conducted stratified random sampling: 30 students were randomly selected from each of the four year groups, comprising 15 males and 15 females per cohort. Paper questionnaires were mailed to the collaborating staff responsible for administration at each institution. Each university received 150 copies, with 120 required to be completed. Collaborators were instructed to promptly replace any damaged or incomplete questionnaires. Through close coordination with university partners, questionnaires were distributed and collected from 2,400 undergraduate students who consented to the recruitment conditions (voluntary participation, anonymity, and confidentiality).

During the data processing stage, this study addressed reverse-scored items within the questionnaire and excluded responses where over 70% of items shared identical answers [35, 36]. Since all four scales used were brief measurement tools (the longest comprising only 12 items), missing values in each scale did not exceed 20, accounting for just 0.9% of observations—well below the 5% limit for missing data [37, 38]. Therefore, median imputation was used at the variable level to handle missing data, maintaining the original distribution while reducing bias in parameter estimates. After applying these methods, 2,340 valid questionnaires were collected, resulting in a response rate of 97.5%. The sample was fairly balanced in terms of gender, with 1,145 males comprising 48.9% and 1,195 females at 51.1%. The grade distribution spanned first- to fourth-year undergraduates with a balanced representation: first-year students (25.6%), second-year students (25.8%), third-year students (24.4%), and fourth-year students (24.2%).

### Research instruments

#### Moral education

This study employed the Moral Education Measurement Scale revised by Chen Shanping et al. to assess university students’ recognition of moral education within physical education classes [4]. The scale comprises three items: ① Emphasis on adhering to sports ethics in PE teaching. ② Inclusion of sports competition rules within PE curriculum content. ③ Teaching content covering regulations and rules in physical education classes. The scale employs a 5-point Likert scale ranging from ‘Strongly Agree’ to ‘Strongly Disagree’, with assigned values from 1 to 5, respectively. All items are reverse-scored. The final scale score is determined by calculating the average score across the three items, with higher scores indicating greater recognition of moral education in physical education classes among university students. Exploratory factor analysis (EFA), KMO = 0.737 > 0.7, and Bartlett’s sphericity test yielded *P* = 0.000 < 0.05. Reliability testing revealed a Cronbach’s α coefficient of 0.918 for the moral education scale, indicating satisfactory reliability and validity.

#### Anomie behaviors in physical education classes

This study employs the Specific Anomie Behavior Scale for University Students in Physical Education Classes developed by Chen Shanping et al. to evaluate students’ anomie behaviors during PE sessions systematically [39] Building upon the original 16-item scale, three items were removed based on dimensional loadings and item-dimension fit, resulting in a final 13-item scale measuring anomie behaviors in physical education classes. The scale encompasses four dimensions: violation of classroom discipline, violation of examination rules, violation of sports ethics, and violation of venue regulations. Items such as ‘Violating classroom discipline during physical education classes,’ ‘Cheating during physical education examinations,’ ‘Demonstrating tenacity and proactive engagement in physical education classes,’ and ‘Deliberately damaging sports equipment and facilities during physical education classes’, reflect behavioral characteristics of university students in physical education classes from different perspectives. The scale employs a five-point Likert scale, with response options ranging from ‘Never’ (1) to ‘Frequently’ (5), corresponding to ‘Rarely’, ‘Seldom’, “Sometimes”, ‘Quite often’, and ‘Frequently’. items 9, 10, and 13 are reverse-scored. The total scale score is derived by calculating the mean of the sixteen items, with higher scores indicating greater frequency of anomie behaviors in sports. The Cronbach’s α coefficient for this scale is 0.801. Confirmatory factor analysis yielded the following results: χ²/df = 14.320, NFI = 0.929, CFI = 0.933, IFI = 0.934, TLI = 0.907, RMSEA = 0.075, CR = 0.801, AVE = 0.640, indicating the scale possesses sound reliability and validity.

#### Attitudes towards sports norms

This study employs the Attitudes Towards Sports Norms developed by Chen Shanping et al. [1] to measure individuals’ value judgments regarding the consequences of complying with sports norms. The scale comprises 20 items across four dimensions: ‘Benefits of Normative Behaviors’, ‘Drawbacks of Normative Behaviors’, ‘Benefits of Anomie Behaviors’, and ‘Drawbacks of Anomie Behaviors’. For example, the statement ‘Adherence to sports norms is beneficial for enhancing physical fitness’ represents a typical expression within the ‘Benefits of Normative Behaviors’ dimension. The scale employs a 5-point Likert scoring method, with response options ranging from ‘Strongly Agree’ to ‘Strongly Disagree’, assigned values from ‘1’ to ‘5’ respectively. Regarding scoring, items within the ‘Benefits of Normative Behaviors’ dimension (Items 1–5) and the ‘Drawbacks of Anomie Behaviors’ dimension (Items 16–20) are reverse-scored. The final total score is calculated as the mean of all items, with higher scores indicating a more positive attitude towards sports norms. In this study, the Cronbach’s α coefficients for the four dimensions were 0.903, 0.877, 0.932, and 0.934, respectively, with an overall scale Cronbach’s α of 0.871. Confirmatory factor analysis yielded the following results: χ²/df = 15.316, NFI = 0.946, CFI = 0.949, IFI = 0.949, TLI = 0.939, RMSEA = 0.078, indicating the scale possesses sound reliability and validity.

#### Self-efficacy in adherence to sports norms

This study employs the Self-Efficacy in Sports Norms Scale developed by Chen Shanping et al. [1]. To assess individuals’ confidence in complying with various behavioral norms during sporting activities. The scale comprises 15 items across four dimensions: rule-governed (6 items), self-consciousness (3 items), benefit-oriented (3 items), and convenience-driven (3 items). For instance, the item ‘I can uphold sports ethics under any circumstances’ reflects an individual’s self-efficacy perception within the rule-governed dimension. The scale employs a five-point Likert scale, with response options ranging from ‘strongly agree’ to ‘strongly disagree’, assigned values from 1 to 5, respectively. All items are reverse-scored. The total scale score is derived by calculating the average of the 15 items, with higher scores indicating stronger self-efficacy regarding sports norms. In this study, the Cronbach’s α coefficients for the scale’s four dimensions were 0.954, 0.869, 0.739, and 0.934, respectively. The overall Cronbach’s α coefficient was 0.939. Confirmatory factor analysis yielded the following results: χ²/df = 10.829 NFI = 0.983, CFI = 0.985, IFI = 0.985, TLI = 0.978, RMSEA = 0.065, indicating the scale possesses sound reliability and validity.

### Research methodology

Data entry and analysis were performed using SPSS 27.0, AMOS 27.0, and PROCESS 4.0 software. The PROCESS macro plugin, developed by renowned statistician Andrew F. Hayes in 2013 [40], is specifically designed for analyzing complex statistical models, with particular strength in testing mediation effects. This plugin efficiently manages intricate structural equation models, effectively streamlining the often cumbersome procedures inherent in traditional statistical analysis. Regarding estimation methods, the PROCESS macro plugin defaults to Bias-Corrected Bootstrap (BC-Bootstrap) interval estimation. BC-Bootstrap is an improved version of the Bootstrap method, enhancing estimation accuracy through bias correction. It is currently considered a stable and reliable computational approach [41].

This study aims to examine the chained mediating role of “attitudes towards sports norms” and “self-efficacy in sports norms” in the relationship between “moral education” and “anomie behaviors in university students’ physical education class”. This model constitutes a multiple mediation framework. To assess its significance, the sixth model within the PROCESS plugin was employed. This model is suitable for analysing sequential multiple mediation effects, enabling simultaneous estimation of direct effects, indirect effects, and the overall effect of the serial mediation. All analyses employed 5,000 Bootstrap resampling iterations to compute 95% confidence intervals. Effects were deemed significant if the interval excluded zero.

This study incorporates gender, grade level, and the distinction between regular students and class officers as control variables in the analysis. Previous research has demonstrated that gender, grade level, and the status of being a regular student versus a class officer exert significant influence on anomie behaviors [42, 4].

## Research findings

### Common method bias test

To mitigate the potential impact of common method bias (CMB) on research findings, this study employed multiple strategies during its design and implementation. Firstly, at the procedural control level, researchers explicitly emphasized confidentiality principles to participants, thereby reducing systematic bias arising from concerns over information leakage. Secondly, in questionnaire design, reverse-scored items were employed and response order optimized to mitigate bias arising from item sequence or directional consistency during completion. Furthermore, to verify the presence and extent of common method bias, Harman’s single-factor test was applied. The results indicated that 11 factors had eigenvalues exceeding 1, with the first factor explaining 12.14% of the variance—below the 40% critical threshold [43]. This demonstrates that the study data exhibit no significant common method bias, permitting progression to subsequent analyses.

### Descriptive statistics and correlation analysis

Descriptive statistics and correlation analyses were conducted on moral education, attitudes towards sports norms, self-efficacy in sports norms, and anomie behaviors in physical education classes. Results indicated significant correlations among all four variables, providing a sound foundation for subsequent mediation effect testing. See Table 1.

**Table 1:** Descriptive statistics and correlation analysis (*n* = 2340)

	M	SD	Moral Education	Attitudes Towards Sports Norms	Self-Efficacy in Sports Norms	Anomie Behaviors in Physical Education Classes
Moral Education	4.289	0.704	1			
Attitudes towards Sports Norms	3.857	0.550	0.364***	1		
Self-Efficacy in Sports Norms	4.609	0.504	0.304***	0.420***	1	
Anomie Behaviors in Physical Education Classes	1.554	0.372	-0.226***	-0.395***	-0.337***	1

### Testing the serial mediation effects of attitudes towards sports norms and Self-Efficacy in sports norms

This study controlled for gender, grade level, and student status (regular students vs. class officers). It examined moral education as the independent variable, anomie behaviors in physical education classes as the dependent variable, and attitudes towards sport norms and sport norms self-efficacy as mediating variables. Serial mediation effects were examined using Model 6 within the PROCESS 3.5 plugin for SPSS developed by Hayes. Before model testing, data underwent standardisation. Bootstrap sampling with 5,000 iterations was employed to calculate 95% confidence intervals, facilitating further testing of the significance of the mediation effect.

**Fig. 2:**
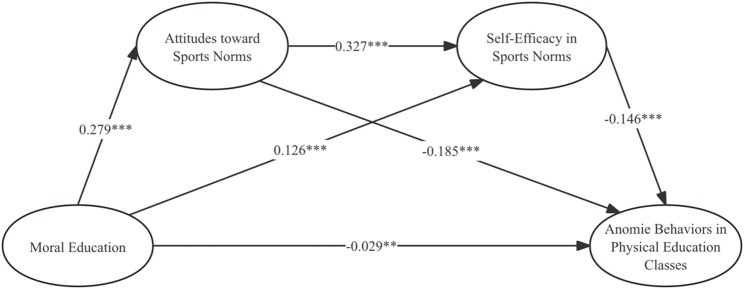
Chain-based intermediary model. * *p*<0.05, ** *p*<0.01, *** *p*<0.001

Regression analysis revealed that moral education significantly and positively predicted attitudes towards sports norms (β = 0.279, t = 18.527, *p* < 0.001) and self-efficacy in sports norms (β = 0.126, t = 8.876, *p* < 0.001), whilst significantly negatively predicting anomie behaviors in physical education classes (β=-0.029, t=-2.740, *p* < 0.01). Furthermore, attitudes towards sports norms significantly and positively predicted self-efficacy in sports norms (β = 0.327, t = 17.897, *p* < 0.001). Conversely, attitudes towards sports norms (β = −0.185, t = − 12.883, *p* < 0.001) and self-efficacy in sports norms (β = -0.146, t = -9.622, *p* < 0.001) significantly and negatively predicted anomie behaviors in physical education classes. See Table 2; Fig. 2 for details.

**Table 2:** Regression analysis of variable relationships

Predictor Variables	Equation 1			Equation 2			Equation 3		
	Attitudes toward Sports Norms			Self-Efficacy in Sports Norms			Anomie Behaviors in Physical Education Classes		
	β	t	95%CI	β	t	95%CI	β	t	95%CI
Gender	-0.078	-3.704***	[-0.119,-0.037]	-0.029	-1.568	[-0.066,0.007]	0.032	2.359*	[0.006,0.060]
grade	-0.041	-4.293***	[-0.059,-0.022]	0.013	1.543	[-0.004,0.030]	0.037	5.914***	[0.025,0.049]
Regular Students and Class officers	-0.039	-1.821	[-0.082,0.003]	-0.008	-0.396	[-0.045,0.030]	0.043	3.092**	[0.016,0.071]
Moral Education	0.279	18.527***		0.126	8.876***	[0.098,0.154]	-0.029	-2.740**	[-0.050,-0.008]
Attitudes toward Sports Norms				0.327	17.897***	[0.292,0.363]	-0.185	-12.883***	[-0.213,-0.157]
Self-Efficacy in Sports Norms							-0.146	-9.622***	[-0.176,-0.117]
R^2^	0.145			0.205			0.212		
F	99.341***			120.139***			104.627***		

The results of the mediation effect analysis indicate that the overall effect of moral education on anomie behaviors in physical education classes is significant (effect value = -0.112, 95% CI = [-0.133, -0.092]), while the direct effect is also significant (effect value = -0.029, 95% CI = [-0.050, -0.008]), indicating that the intervening variables partially mediate the influence of moral education on anomie behaviors in physical education classes. Although the standardized coefficients (β) observed in this study fall within the range of small to moderate effects—a common and expected outcome in social behaviors research involving multiple influencing factors—the practical significance of these effects should not be overlooked. First, the overall impact of moral education on anomie behaviors in physical education classes is mediated by attitudes toward sports norms and self-efficacy, accounting for as much as 74.1% of the total effect. This finding holds clear practical implications: it suggests that interventions aimed at reducing anomie behaviors in physical education classes would be more effective if they shifted focus from mere rule enforcement to enhancing moral education. This approach would shape students’ attitudes toward sports norms and boost their self-efficacy in adhering to them. Second, from a group-level perspective, implementing moral education across the entire school would yield significant positive behavioral improvements overall.

The mediation effect comprises three pathways: Path 1 (moral education → attitude towards sports norms →anomie behaviors in physical education classes) yielded an effect size of -0.051, 95% CI = [-0.062, -0.042], confirming the pathway; Path 2 (moral education → self-efficacy in sports norms → anomie behaviors in physical education classes) yielded an effect size of -0.018, 95% CI = [-0.025, -0.012], confirming the path; Path 3 (moral education → attitude towards sports norms → self-efficacy in sports norms →anomie behaviors in physical education classes) yielded an effect size of -0.013, with a 95% CI of [-0.018, -0.010], confirming the path. The confidence intervals for all three mediating paths excluded zero, indicating that attitudes towards sports norms and self-efficacy in sports norms mediated the relationship between moral education and anomie behaviors in physical education classes (with Path 3 representing serial mediation). See Table 3 for details.

**Table 3:** Mediation effect test

	Effect Value	SE	LLCI	ULCI	Mediation Proportion
Total Effect	-0.112	0.011	-0.133	-0.092	100.00%
Direct Effect	-0.029	0.011	-0.050	-0.008	25.89%
total indirect effect	-0.083	0.007	-0.097	-0.070	74.11%
Path 1	-0.051	0.005	-0.062	-0.042	45.54%
Path 2	-0.018	0.003	-0.025	-0.012	16.07%
Path 3	-0.013	0.002	-0.018	-0.01	11.61%

## Discussion

### The impact of moral education on anomie behaviors in physical education classes

This study’s findings indicate a significant negative correlation between moral education and Anomie behaviors in physical education classes, with moral education exerting a direct and significant effect on such anomie behaviors in physical education classes. Overall, the depth of moral education exhibits an inverse relationship with the frequency of anomie behaviors in physical education classes. Relevant studies indicate that a more favorable moral climate correlates with fewer instances of anomie behaviors [44]; a robust moral atmosphere serves as a latent educational force, propelling the elevation of individuals’ moral cognition, moral sentiments, and moral behaviors [45]; an ethical ethos can constrain athletes to adhere to rules and cultivate their ideological and moral character, thereby reducing the occurrence of anomie behaviors [46], fostering steadfast ethical characteristics in sports can effectively diminish instances of anomie behaviors [47]. Systematic and contextually relevant moral education within sporting settings can explicitly inform students about hazardous activities, malicious conduct, and harmful actions during physical activities [48], applying pressure to deter such misconduct and intervening promptly [49]. This direct transmission of norms effectively reduces the occurrence of anomie behaviors during physical education classes. Furthermore, sports moral education helps students develop self-discipline, personal principles, and a sense of moral boundaries. By continuously monitoring the rate of transfer and transformation during this development, students can rapidly assess the legitimacy of their actions, directly constraining their behaviors and reducing the likelihood of anomie behaviors occurring [50].

The significant direct effect observed between moral education and anomie behaviors in physical education classes provides clear empirical evidence for understanding their relationship. From the perspective of social cognitive theory [34] moral education, as a key external environmental factor, can exert a direct guiding influence on individuals’ cognitive processes and behavioral choices by systematically conveying socially recognised behavioral codes and ethical norms. As emphasised by Bandura’s triadic reciprocal determinism model [30], when moral education content is sufficiently concrete, clear, and robustly implemented, it inherently functions as a potent behavioral guide. Under such conditions, it can directly inhibit anomie behaviors without necessarily requiring complex intermediate psychological mechanisms to exert its regulatory effect. As relevant research indicates, when moral education content is closely integrated with physical education practice, it can directly influence the realm of social life [51], forming direct norms for individual behavior [52], and directly impacting students’ behaviors in physical education classes. This confirms the significant direct effect of moral education on anomie behaviors in physical education classes. This finding demonstrates that strengthening the direct guiding role of moral education is an effective pathway for improving anomie behaviors in physical education classes.

### Explanation of the independent mediating effects of attitudes towards sports norms and self-efficacy in sports norms

The findings reveal that attitudes towards sports norms exert a significant mediating effect between moral education and anomie behaviors in university physical education classes. This discovery aligns with Attitude-Behavior Theory [17], which posits that external education indirectly influences actual behavioral performance by shaping individuals’ attitudinal inclinations towards normative behaviors. Within university physical education classes, cultivating positive attitudes towards sports norms helps reduce anomie behaviors from an intrinsic level. Durkheim proposed that moral education provides explicit moral rules and behavioral standards, requiring individuals to learn rule observance, respect for rules, and compliance with norms [53], thereby effectively reducing anomie behaviors in sports and playing a vital role in personal development [54]. According to Kelman’s attitude formation theory, moral education constitutes a purposeful, planned, and organised social practice that employs specific ideological and political perspectives alongside ethical norms to influence university students. This process fosters the development of the moral character demanded by society [55], reinforcing positive behavioral attitudes and consequently reducing the frequency of deviant conduct in physical education classes.

Furthermore, positive behavioral attitudes can significantly reduce the likelihood of rule-breaking, with behavioral attitude variables influencing university students’ intentions towards moral deviance [56]. Consequently, moral education serves to predict and explain the formation of students’ attitudes towards sports norms. It inhibits the impulse to breach norms at an intrinsic cognitive level, thereby effectively reducing the occurrence of anomie behaviors in sports.

Concurrently, self-efficacy in sports norms regarding also exhibits an independent mediating effect between moral education and anomie behaviors in university physical education classes. According to Bandura’s social cognitive theory, the development of self-efficacy may be influenced by the interplay of multiple factors, including social support, school adaptation, and individual coping strategies [57]. Should institutions implement systematic moral education guided by self-efficacy principles to reinforce students’ comprehension of sports norms. This cognitive process enables learners to recognise their capacity to acquire specific moral standards, master relevant skills for upholding sports regulations, and practise certain moral behaviors. It also fosters self-discipline in sports class behaviors. Furthermore, existing research suggests that self-efficacy serves as a mediating factor, influencing individuals’ cognitive patterns and thereby indirectly shaping their behaviors [58]. Consequently, moral education that enhances university students’ self-efficacy in sports norms can prompt them to recognise the significance of these norms more clearly and approach anomie behaviors with greater confidence. This, in turn, fosters an internalised reinforcement of self-regulation during physical education classes, holding significant practical implications for mitigating anomie behaviors among university students in such settings.

### Explaining the serial mediation effects of attitudes towards sports norms and Self-Efficacy in sports norms

This study innovatively reveals the serial mediation role of attitudes toward sports norms and self-efficacy in influencing the effect of moral education on anomie behaviors in university physical education classes. By incorporating the full chain of ‘attitude formation → efficacy-driven behavior,’ this approach substantiates the comprehensive influence mechanism proposed in the introduction, thereby addressing limitations of previous research that poorly analyzed multiple mediating pathways. Specifically, moral education first guides individuals to develop positive attitudes toward sports norms, which in turn boosts their self-efficacy in sports norms and ultimately reduces the occurrence of anomie behaviors in university physical education classes. In other words, stronger moral education leads to more positive attitudes toward sports norms, greater self-efficacy in sports norms, and a lower rate of anomie behaviors in university physical education classes. This process aligns with the core pathway proposed by Ajzen [17], the Theory of Planned Behavior – ‘Attitude/Subjective Norm/Perceived Behavioral Control → Behavioral Intention → Actual Behavior’ – both confirming the universality of multiple variables on behavior and emphasizing the synergistic effects of mediating factors. Past research has predominantly focused on single mediators. By considering dual mediation, this study uncovers how moral education influences anomie behaviors through a combined mechanism. This offers a dynamic systems perspective on how educational interventions profoundly impact individual behavior and sets the stage for future research exploring parallel multi-mediation pathways.

However, from the overall theoretical and empirical research context, the relationship between attitudes towards sports norms and self-efficacy is not unidirectional or irreversible. The behavioral success experiences derived from enhanced self-efficacy in sports norms may conversely reinforce individuals’ positive attitudes towards sports norms. For instance: students with higher academic self-efficacy exhibit more positive learning attitudes [59]. Secondary school students with higher self-efficacy tend to exhibit more positive attitudes towards physical exercise [60]. Possessing high self-efficacy—that is, strong confidence in one’s ability to accomplish actions—generates positive behavioral attitudes [61].

## Conclusions and limitations

This study reveals the intrinsic pathways through which moral education influences anomie behaviors in university physical education classes, providing empirical evidence that moral education optimizes classroom conduct. The conclusions are as follows: (1) Moral education has a negative predictive effect on anomie behaviors in physical education classes. This finding provides empirical support for the notion that moral education optimizes classroom conduct in physical education settings. (2) Attitudes toward sports norms and self-efficacy in sports norms independently mediate the impact of moral education on anomie behaviors in university physical education classes. This indicates that moral education can reduce anomie behaviors in physical education classes both by enhancing attitudes toward sports norms and by strengthening self-efficacy in sports norms. (3) Attitudes toward sports norms and self-efficacy in sports norms play a serial mediation role in connecting moral education with anomie behaviors in physical education classes. Moral education not only directly reduces anomie behaviors in physical education classes but does so progressively by first fostering positive attitudes toward sports norms among students, thereby enhancing their self-efficacy in sports norms, and ultimately reducing anomie behaviors in physical education classes.

### Recommendations

 (1) In practice, we should promote the organic integration of moral education and physical education, embedding moral education systems into physical education classes. Through contextualized and case-based instructional design, students should be guided to understand normative values within authentic athletic scenarios, thereby effectively reducing anomie behaviors in physical education classes. (2) Emphasize dual-pathway interventions: on one hand, deepening students’ attitudes toward sports norms through moral discussions and exemplary role models; on the other hand, strengthening their confidence and ability to adhere to sports norms through tiered goal-setting and timely feedback. (3) Teaching practices should design sequential instructional activities following a progressive path of “value recognition—competency development—behavioral consolidation”: First, deepen students’ understanding of sports norms through contextual discussions. Next, create tiered practical tasks to help students build confidence in their abilities through successful experiences. Finally, facilitate the stable internalization of normative behaviors.

Furthermore, the findings of this study provide a micro-level empirical foundation for understanding the role of physical education curricula within China’s contemporary strategies of “building an education powerhouse” and “cultivating virtue through education.” Findings indicate that physical education serves as an effective vehicle for achieving the policy goal of “cultivating character through physical education.” Its educational efficacy is not merely theoretical but is realized through observable, intervenable psychological mechanisms—specifically, attitudes toward sports norms and self-efficacy in sports norms. This suggests that educational administrators should move beyond a purely skill-oriented approach in curriculum design and assessment. Instead, they should incorporate the cultivation of students’ attitudes toward sports norms and self-efficacy as core objectives, thereby implementing macro-level educational policies at the classroom level.

### Limitations and future prospects

This study has limitations. First, In terms of research design, this study employs cross-sectional survey data. While this approach can statistically reveal patterns of association and mediating pathways among variables, it cannot rigorously establish the direction of causality or temporal dynamics between variables. For instance, moral education may reduce anomie behaviors in physical education classes, but it is also possible that students with better behavioral norms are more inclined to agree with the content of moral education. Reverse causality or confounding by third variables may be present. Future research could employ longitudinal tracking designs to measure core variables at multiple time points, or conduct randomized controlled trials of moral education interventions. This would allow for more rigorous examination of the causal effects of moral education on attitudes toward sports norms, self-efficacy in sports norms, and anomie behaviors in physical education classes. Secondly, regarding data sources, all variables in this study primarily rely on student self-report questionnaires, potentially introducing common method bias. Furthermore, self-reporting of “anomie behaviors in physical education classes” may be influenced by social desirability, potentially underestimating actual occurrence rates. Future studies could integrate multi-source data—such as teacher evaluations of student classroom behavior, peer assessments, or classroom observation records—to enhance objectivity and convergent validity, thereby providing a more comprehensive and accurate portrayal of actual anomie behaviors in physical education classes. Finally, regarding mechanism exploration, this study preliminarily validated the chained mediating pathway of “attitudes toward sports norms → self-efficacy in sports norms”. However, this mechanism is likely dynamic and bidirectional. Attitudes toward sports norms may enhance self-efficacy in sports norms, while successful self-efficacy in sports norms may in turn reinforce attitudes toward sports norms, suggesting a mutually reinforcing relationship. Constrained by cross-sectional data, this study could not examine this reciprocal relationship. Future research utilizing longitudinal data from multiple measurements, employing analytical methods such as cross-lagged panel models, could delve deeper into the interactive trajectories between attitudes toward sports norms and self-efficacy in sports norms over time, thereby providing a more complete understanding of their co-evolutionary mechanisms.

## Supplementary Information

Supplementary Material 1

## Data Availability

The data and materials of this study were collected and collated by the authors and used to support the conclusions of this paper, and the authors have no undue reservations.
